# Ferrichrome, a fungal-type siderophore, confers high ammonium tolerance to fission yeast

**DOI:** 10.1038/s41598-022-22108-0

**Published:** 2022-10-27

**Authors:** Po-Chang Chiu, Yuri Nakamura, Shinichi Nishimura, Toshitsugu Tabuchi, Yoko Yashiroda, Go Hirai, Akihisa Matsuyama, Minoru Yoshida

**Affiliations:** 1 Department of Biotechnology, Graduate School of Agricultural and Life Sciences, The University of Tokyo, Tokyo, 113-8657 Japan; 2 Collaborative Research Institute for Innovative Microbiology, The University of Tokyo, Tokyo, 113-8657 Japan; 3 RIKEN Center for Sustainable Resource Science, Wako, Saitama 351-0198 Japan; 4 Graduate School of Pharmaceutical Sciences, Kyushu University, 3-1-1 Maidashi, Higashi-ku, Fukuoka 812-8582 Japan

**Keywords:** Fungi, Natural products

## Abstract

Microorganisms and plants produce siderophores, which function to transport environmental iron into cells as well as participate in cellular iron use and deposition. Their biological functions are diverse although their role in primary metabolism is poorly understood. Ferrichrome is a fungal-type siderophore synthesized by nonribosomal peptide synthetase (NRPS). Herein we show that ferrichrome induces adaptive growth of fission yeast on high ammonium media. Ammonium is a preferred nitrogen source as it suppresses uptake and catabolism of less preferred nitrogen sources such as leucine through a mechanism called nitrogen catabolite repression (NCR). Therefore, the growth of fission yeast mutant cells with leucine auxotrophy is suppressed in the presence of high concentrations of ammonium. This growth suppression was canceled by ferrichrome in a manner dependent on the amino acid transporter Cat1. Additionally, growth retardation of wild-type cells by excess ammonium was exacerbated by deleting the NRPS gene *sib1*, which is responsible for the biosynthesis of ferrichrome, suggesting that intrinsically produced ferrichrome functions in suppressing the metabolic action of ammonium. Furthermore, ferrichrome facilitated the growth of both wild-type and *sib1*-deficient cells under low glucose conditions. These results suggest that intracellular iron regulates primary metabolism, including NCR, which is mediated by siderophores.

## Introduction

**Figure 1:**
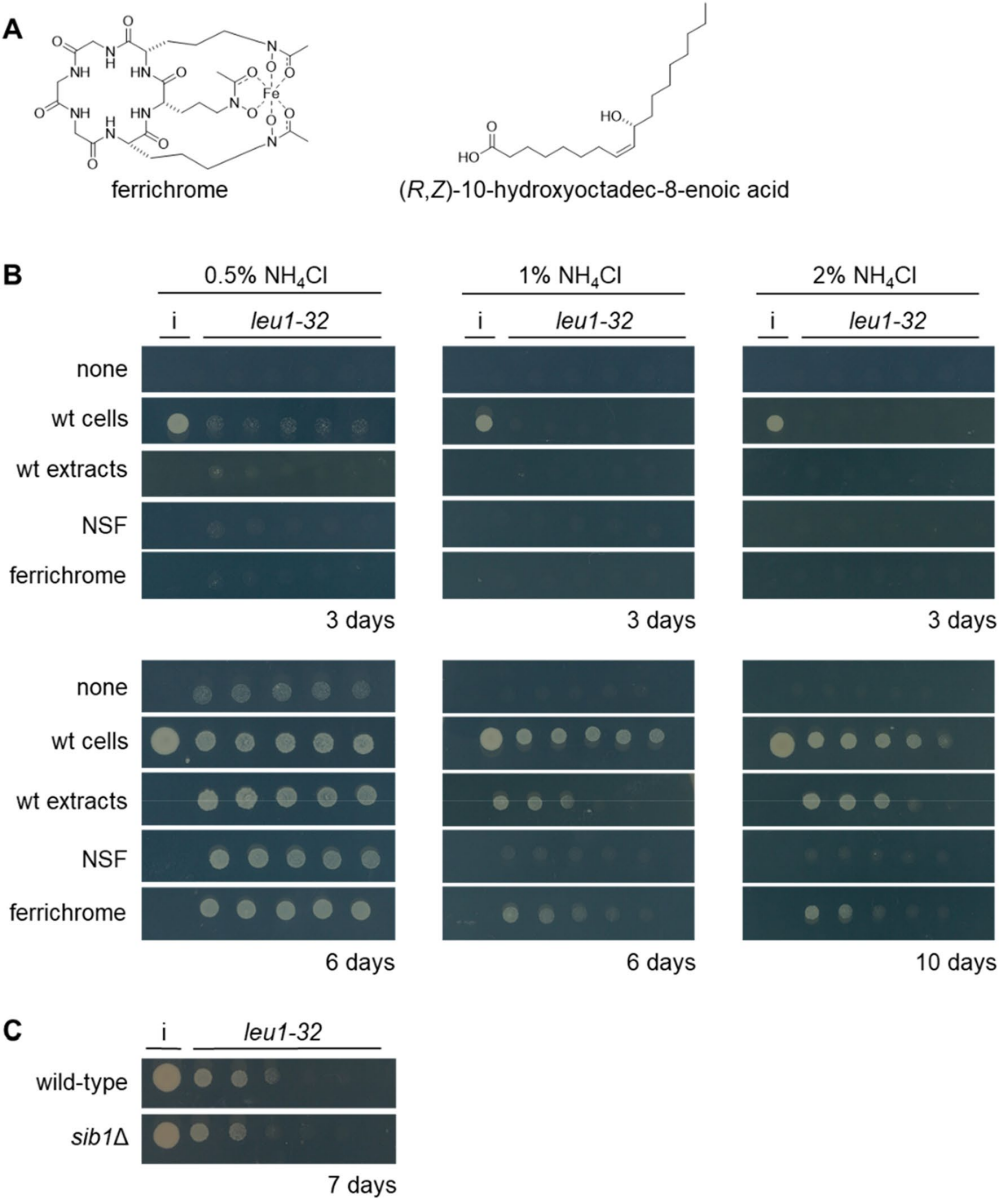
Ferrichrome induces the growth of leucine-auxotrophic cells on high ammonium media. (**A**) Chemical structures of ferrichrome and (*R,Z*)-10-hydroxyoctadec-8-enoic acid (used as an NSF in this study). (**B**) Adaptive growth of leucine-auxotrophic *leu1-32* cells by wild-type inoculation or chemicals. A cell suspension of the *leu1-32* strain was spotted on a variety of EMM plates containing 0.5%, 1.0% or 2.0% NH_4_Cl and 0.2 mM of leucine as nitrogen sources. Wild-type cell suspension (3 μl of a 0.2 OD_595_ suspension), cell extract (3 μl of a 250 μg/ml extract), NSF (150 ng in 3 μl 50% MeOH) or ferrichrome (150 ng in 3 μl of 50% MeOH) was spotted to the left of the five spots of *leu1-32* cells. Representative images from three independent experiments are shown. (**C**) Wild-type or *sib1*Δ cells were used as an inducer strain. Cells were incubated on EMM containing 2.0% NH_4_Cl and leucine, supplemented with dipyridyl (100 μM). Dipyridiyl was added to induce production of ferrichome.

Nonribosomal peptide synthetase (NRPS) is one of the major machineries that synthesizes secondary metabolites^1^. Siderophores are iron-chelating metabolites of microbes and plants, most of which are synthesized by NRPS^2,3^. The molecular function of siderophores is to simply chelate iron. However, their biological functions are diverse as they act not only as an iron courier under iron-starved conditions, but also as metallophores for a variety of metals^4^, social communication molecules of microbes^5,6^, virulence factors for pathogens^7^, and antibiotics known as sideromycins^8,9^. Ferrichromes, a major class of fungal siderophores, are cyclic hexapeptides containing three residues of *N*-acyl-*N*-hydroxyornithine in tandem by which they chelate iron (Fig. 1A)^10^. They usually reside in cells to sequester iron^11,12^. The genome of the fission yeast *Schizosaccharomyces pombe* encodes the NRPS Sib1, which synthesizes ferrichrome^13^.

In fission yeast, ferrichrome is mostly stored in cells^14^ and its biosynthesis is increased under iron-depleted conditions^15^. Mutants lacking ferrichrome show defects in growth under iron-depleted conditions^15^ and in spore germination^16^. Thus, ferrichrome seems to contribute to the acquisition of exogenous iron under iron-limiting conditions. Fission yeast has a siderophore transporter Str1, by which exogenously supplied ferrichrome can be used^16,17^. However, the biological functions and the metabolism of ferrichrome largely remain to be elucidated.

Ammonium is an excellent nitrogen source for many microorganisms^18–20^. Cells exhibit preferred uptake of high-quality nitrogen sources such as ammonium and glutamate, which results in the rapid cellular biosynthesis of nitrogen-containing metabolites. The presence of preferred nitrogen sources suppresses incorporation and catabolism of less preferred nitrogen sources such as branched chain amino acids (BCAAs; valine, leucine, and isoleucine), a process which is called nitrogen catabolite repression (NCR). The fission yeast *S. pombe* prefers ammonium or glutamate as its sole nitrogen source^21,22^. The presence of ammonium suppresses uptake of leucine from the medium^23^. The inhibition of leucine uptake was not observed by deleting the *pub1* gene, which encodes the E3 ubiquitin-protein ligase for membrane amino acid transporters^24,25^. Cells lacking the *eca39* gene (*eca39*Δ), which encodes an aminotransferase for synthesizing BCAA, and leucine-auxotrophic *leu1-32* mutant cells are unable to grow on media containing NH_4_Cl or glutamate in spite of supplementation with BCAA^26,27^.

Surprisingly, fission yeast cells adapt to NCR as indicated by the fact that BCAA-auxotrophic cells show adaptive growth in the vicinity of growing cells^26,27^. The adapted *eca39*Δ cells reprogram transcription, which showed high correlations with those of histone-modifying enzyme mutants such as *gcn5*Δ. The Agp3 amino acid transporter is the putative downstream effector of Gcn5 since *agp3* knockout suppresses NCR tolerance of *gcn5*Δ cells. Molecules secreted by wild-type cells to induce adaptive growth were identified to be oxylipins that are referred to as nitrogen signaling factors (NSFs) and include (*R,Z*)-10-hydroxyoctadec-8-enoic acid and its acetylated derivative (Fig. 1A)^27,28^. Notably, *agp3* is required for the action of NSF, thus NSF, Gcn5 and Agp3 likely function in the same pathway.

In this study, we demonstrate that ferrichrome, a fission yeast siderophore, induces adaptive growth of leucine-auxotrophic cells on high ammonium media. In contrast to the case of NSF, Agp3 was not required for the action of ferrichrome, while growth suppression by high glutamate was not canceled by ferrichrome. Additionally, ferrichrome-deficient cells without leucine auxotrophy showed higher sensitivity to ammonium than the wild-type cells. Our results indicate that ferrichrome antagonizes the action of ammonium and suppresses the NCR, suggesting that there is functional crosstalk between iron and ammonium metabolism.

## Results and discussion

### Identification of ferrichrome as a metabolite that supports adaptive growth

Cells with leucine auxotrophy (*leu1-32* strain) show weak growth on the synthetic medium EMM supplemented with 0.2 mM leucine (Fig. 1B). EMM contains 0.5% NH_4_Cl. No visible growth was observed on EMM containing higher concentrations of NH_4_Cl, i.e., 1.0% and 2.0% NH_4_Cl that are two and four times higher than the NH_4_Cl concentration in regular EMM, respectively. When wild-type cells were inoculated next to *leu1-32* mutant cells, the auxotrophic cells showed adaptive growth. As the concentration of NH_4_Cl increased, a longer incubation time was required for colony formation. Colony formation was observed after six days on 0.5% or 1.0% NH_4_Cl medium, while it took 10 days on 2.0% NH_4_Cl medium. Wild-type cells may excrete molecules that induce adaptive growth. NSF is a known adaptive growth inducer that is detected in the culture supernatant of fission yeast. NSF induced adaptive growth, but it was effective only on regular EMM. When cell extracts of the wild-type strain were used, adaptive growth was observed for all of the conditions tested. To identify the adaptive growth inducer in the cell lysate, bioassay-guided fractionation was conducted. Active fractions were shown to contain a metabolite that had UV/Vis absorption at 425 nm from the HPLC analyses (Fig. S1). This characteristic absorption suggested that the metabolite was an iron-chelating metabolite, such as ferrichrome (Fig. 1A**)**^13,14^. We compared the HPLC profiles of the extract-derived metabolite and the authentic ferrichrome to confirm that the two substances were identical (Fig. S1). As expected, we observed adaptive growth by ferrichrome (Fig. 1B), which led us to conclude that ferrichrome was one of the metabolites supporting the adaptive growth of the leucine-auxotrophic strain under high NH_4_Cl conditions. This does not rule out the possibility that adaptive growth inducers other than NSF or ferrichrome could be excreted from fission yeast cells. In fact, cells lacking *sib1*, which do not produce ferrichrome, induced adaptive growth (Fig. 1C).

**Figure 2:**
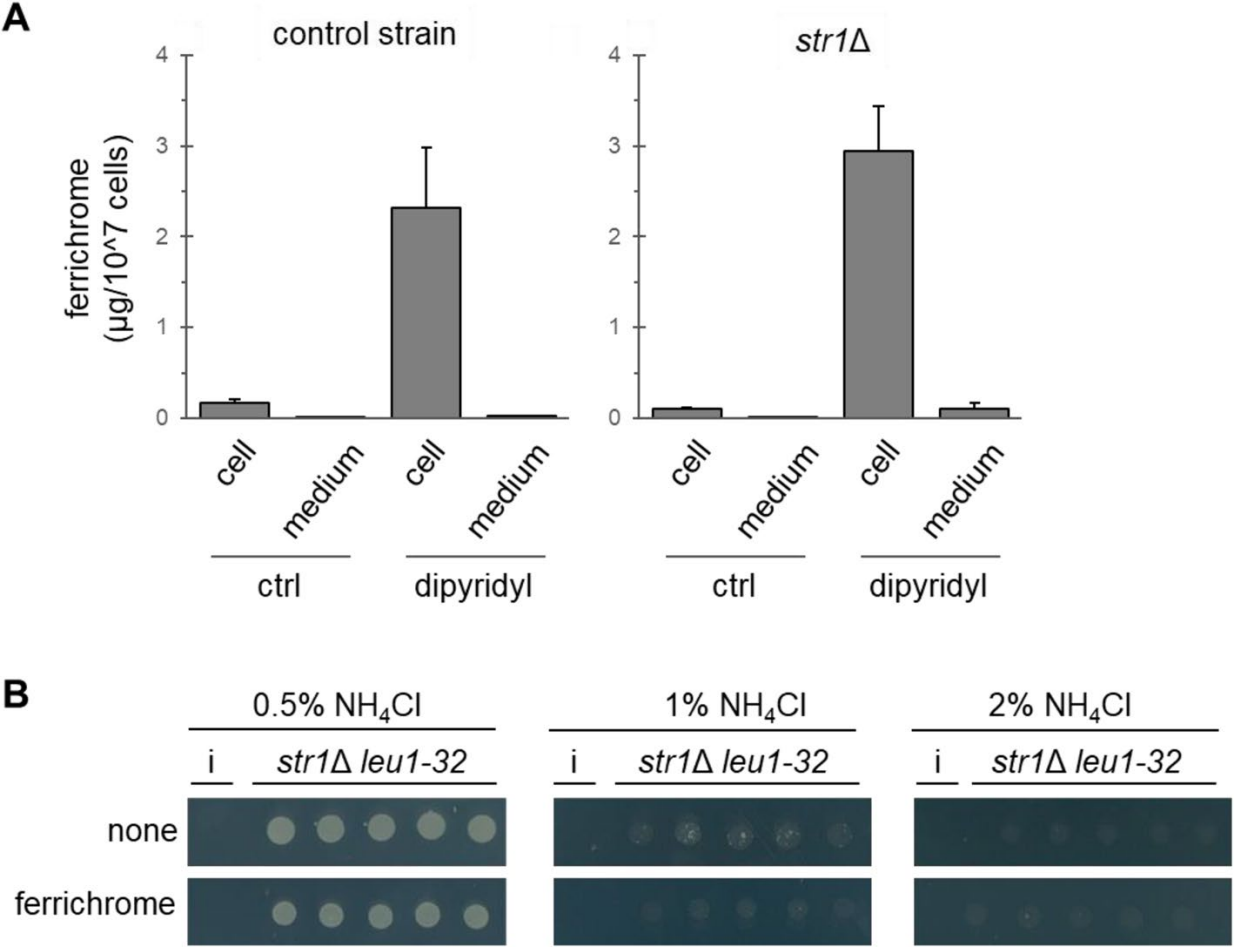
Intracellular localization and function of ferrichrome. (**A**) Quantitation of ferrichrome in cells or media. Cells were incubated at 27 °C for 24 h with or without dipyridyl (100 μM). Dipyridiyl is an iron chelator that induces iron starvation. The amount of ferrichrome in cells and culture media was quantified by HPLC. Ferrichrome was not detected in the *sib1*Δ cells or the medium. Data represent the mean ± SE (*n* = 3). (**B**) Effect of ferrichrome on *str1*Δ *leu1-32* cells. Cells were cultivated at 30 °C for 5 days on EMM supplemented with leucine. Concentrations of NH_4_Cl were 0.5%, 1.0% or 2.0%. A sample of 50% MeOH (3 µl, shown as none) or ferrichrome (150 ng in 3 µl of 50% MeOH) was spotted on the left of the yeast suspensions. Representative images from three independent experiments are shown.

Ferrichrome was reported to reside in *S. pombe* cells^14^. We cultivated fission yeast in liquid media, examined the distribution, and confirmed the previous results that more than 95% of ferrichrome was detected in cells and less than 5% in the culture supernatant (Fig. 2). The production was drastically increased under iron-starved conditions where more than 95% of ferrichrome was detected in cells. *S. pombe* has one plasma membrane siderophore transporter Str1^16,17^. We examined the possibility that ferrichrome is excreted into the medium and then incorporated through Str1. Cells lacking *str1* showed a similar distribution of ferrichrome with that of wild-type cells (Fig. 2A), indicating that the amount of ferrichrome in the medium is small, regardless of the presence or absence of *str1*. Importantly, *str1*Δ cells did not show adaptive growth by ferrichrome (Fig. 2B). These results indicated that the adaptive growth induced by ferrichrome was mediated by its uptake into the cells through a specific ferrichrome transporter and it was not excreted.

We previously identified NSFs in the culture supernatant of prototrophic cells^27^. To investigate if NSF and ferrichrome function in the same pathway, we compared their effects using two experiments. First, we examined the effects of the nitrogen sources. We confirmed that NSF induces adaptive growth of leucine-auxotrophic cells on the medium containing NH_4_Cl (Fig. 1B) and BCAA-auxotrophic *eca39*Δ cells on high glutamate medium (Fig. S2A). In contrast, ferrichrome did not support the growth of *eca39*Δ cells on high glutamate medium. We next tested mutant cells lacking the *agp3* gene, which codes for an amino acid transporter that is required for the action of NSF^27^. NSF did not induce the adaptive growth of *agp3*Δ cells, while ferrichrome did induce the adaptive growth of *agp3*Δ cells (Fig. S2B). These results indicated that the action of ferrichrome was different from that of NSF.

### Involvement of the Cat1 amino acid transporter in the adaptive growth of leucine-auxotrophic cells

**Figure 3:**
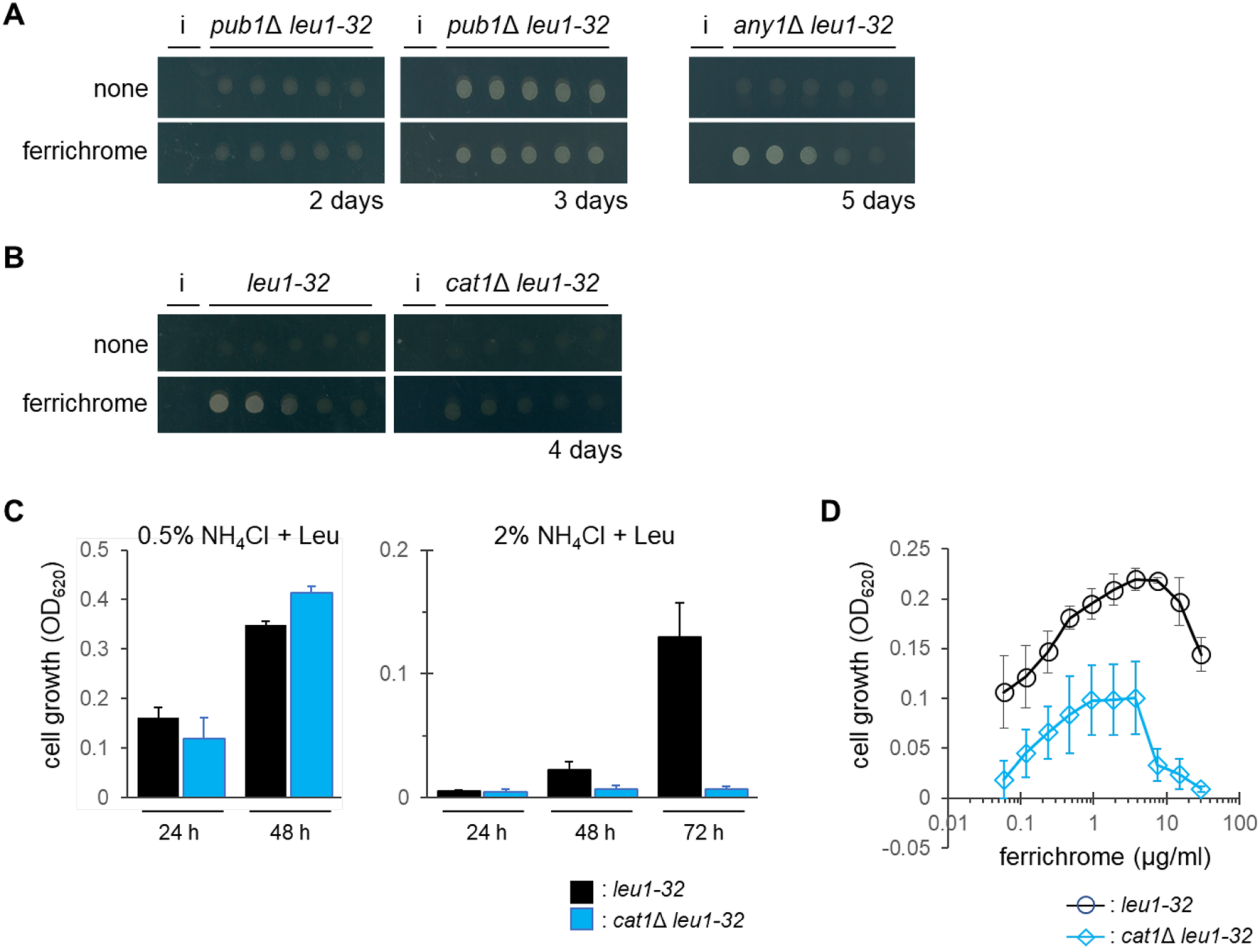
Involvement of amino acid transporters in the action of ferrichrome. (**A**) Growth of *pub1*Δ *leu1-32* and *any1*Δ *leu1-32* cells on EMM containing 2.0% NH_4_Cl supplemented with leucine. A sample of 50% MeOH (3 μl) or ferrichrome (150 ng in 3 μl 50% MeOH) was spotted next to the yeast cell suspensions (denoted as ‘i’). Representative images from three independent experiments are shown. (**B**) Growth of *leu1-32* and *cat1*Δ *leu1-32* cells on EMM containing 2.0% NH_4_Cl supplemented with leucine. Adaptive growth was observed as in A. Representative images from three independent experiments are shown. (**C**) Effect of *cat1* deletion on the cell growth in liquid media. Cells were inoculated in EMM + 2.0% NH_4_Cl supplemented with leucine. Data represent the mean ± SD (*n* = 3). (**D**) Effect of *cat1* deletion on the growth recovery by ferrichrome. Cells were inoculated as in C for 48 h with a variety of concentrations of ferrichrome. Data represent the mean ± SD (*n* = 3–4).

To investigate the molecular mechanism of growth recovery by ferrichrome, we tested two mutant cells lacking the *pub1* and *any1* genes. Pub1 and Any1 regulate the cellular localization of amino acid transporters such as Aat1 and Cat1^24,25,29^. In the *pub1*Δcells, the transporters are mostly localized at the plasma membrane, while some portions of transporters remain at intracellular organelles in the *any1*Δ cells^24^. The effect of knockout of *pub1* was observed for the* pub1*∆ *leu1-32* cells, which grew well, and was independent of the addition of ferrichrome (Fig. 3A). This can be explained by the increased uptake of leucine in *pub1*Δ cells^23^. In contrast, the *any1*∆ *leu1-32* cells exhibited growth induction in the presence of added ferrichrome (Fig. 3A). Based on these results, amino acid transporters whose localization is mainly regulated by Pub1 seem more likely to be involved in adaptive growth by ferrichrome.

To identify the transporters responsible for the action of ferrichrome, we tested five independent mutants, each lacking an amino acid transporter (Fig. S3). Among five mutants tested, poorer growth recovery was observed for the *cat1*Δ *leu1-32* cells. The adaptive growth of *leu1-32* cells by ferrichrome was dependent on *cat1* (Fig. 3B). The *cat1* gene encodes the plasma membrane arginine/lysine amino acid transmembrane transporter Cat1. Quantitative analyses in the liquid media showed that the growth of the *cat1*Δ *leu1-32* cells was comparable to that of the parental *leu1-32* cells in 0.5% NH_4_Cl medium, while the growth was severely suppressed in 2.0% NH_4_Cl medium than that of the control cells (Fig. 3C). Growth recovery of the parental strain by ferrichrome was also detected in liquid media, which was observed at the lowest concentration tested (0.059 μg/ml; 85.8 nM) and in a concentration-dependent manner (Fig. 3D). Higher concentrations (more than 7.5 μg/ml) were less effective, probably because of the disruption of iron homeostasis. *cat1*Δ *leu1-32* cells showed growth recovery by ferrichrome, but it was much less significant than the recovery observed for the control strain. These results suggest that Cat1 is one of the amino acid transporters responsible for leucine uptake when *leu1-32* cells show adaptive growth by ferrichrome under high ammonium conditions. However, given that the *cat1*Δ *leu1-32* cells still weakly responded to ferrichrome (Fig. 3C), other leucine transporters should also be involved in the ferrichrome-induced adaptive growth. In addition, we cannot exclude the possibility that *cat1*Δ *leu1-32* cells produce a lower amount of endogenous ferrichrome than *leu1-32* cells.

### Ferrichrome-deficient cells are less tolerant to high ammonium levels

**Figure 4:**
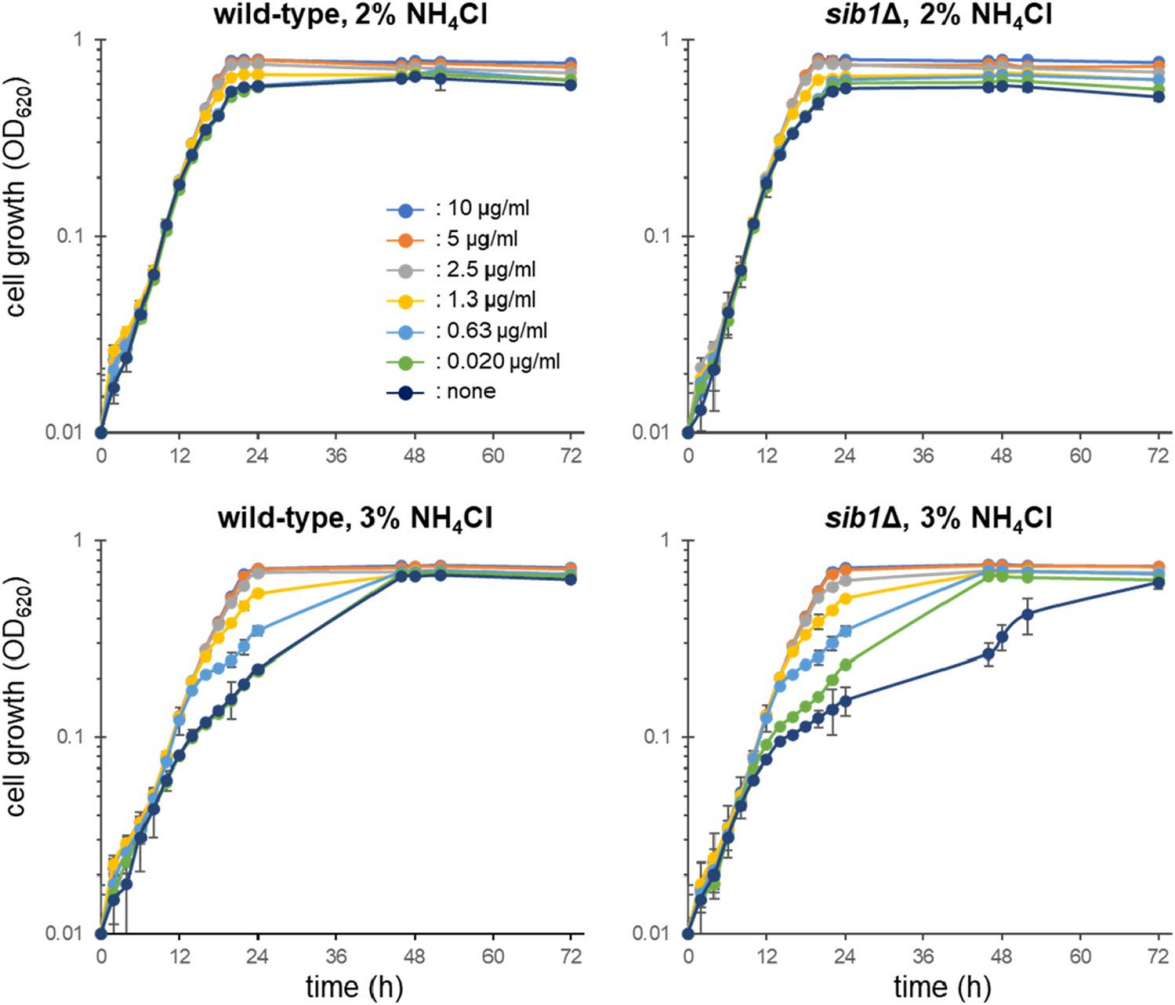
Growth of wild-type and *sib1*Δ cells in high ammonium media. Comparison of the wild-type and *sib*1∆ mutant cells in EMM containing 2.0% or 3.0% NH_4_Cl with various concentrations of ferrichrome. Concentrations of ferrichrome ranged from 0 to 10 µg/ml. Data represent the mean ± SD (*n* = 3).

Exogenous ferrichrome induced adaptive growth as described above, while endogenous ferrichrome is mainly localized within wild-type cells (Fig. 2). We next investigated if ferrichrome-deficient cells, such as *sib1*Δ cells, show growth retardation under high ammonium conditions (Fig. 4). Wild-type and *sib1*Δ cells showed comparable growth in 2.0% NH_4_Cl medium. Addition of exogenous ferrichrome resulted in only a slight effect on the growth of both strains. In 3.0% NH_4_Cl medium, the growth speed of both strains became slower after exposure to the high NH_4_Cl condition for 14 h. The slope of *sib1*Δ cell growth after 14 h was more gradual than that of the wild-type cells (Fig. 4). Addition of ferrichrome resulted in a significant effect on the growth in 3.0% NH_4_Cl medium. Growth of both strains in 3.0% NH_4_Cl medium with 2.5–10 μg/ml of ferrichrome was comparable to that in 2.0% NH_4_Cl medium. The lowest effective concentration of ferrichrome was 20 ng/ml (28.4 nM), a concentration at which the *sib1*Δ cells had comparable growth to that of wild-type cells.

### Ferrichrome is still effective under iron-rich conditions, and less effective under low glucose conditions

**Figure 5:**
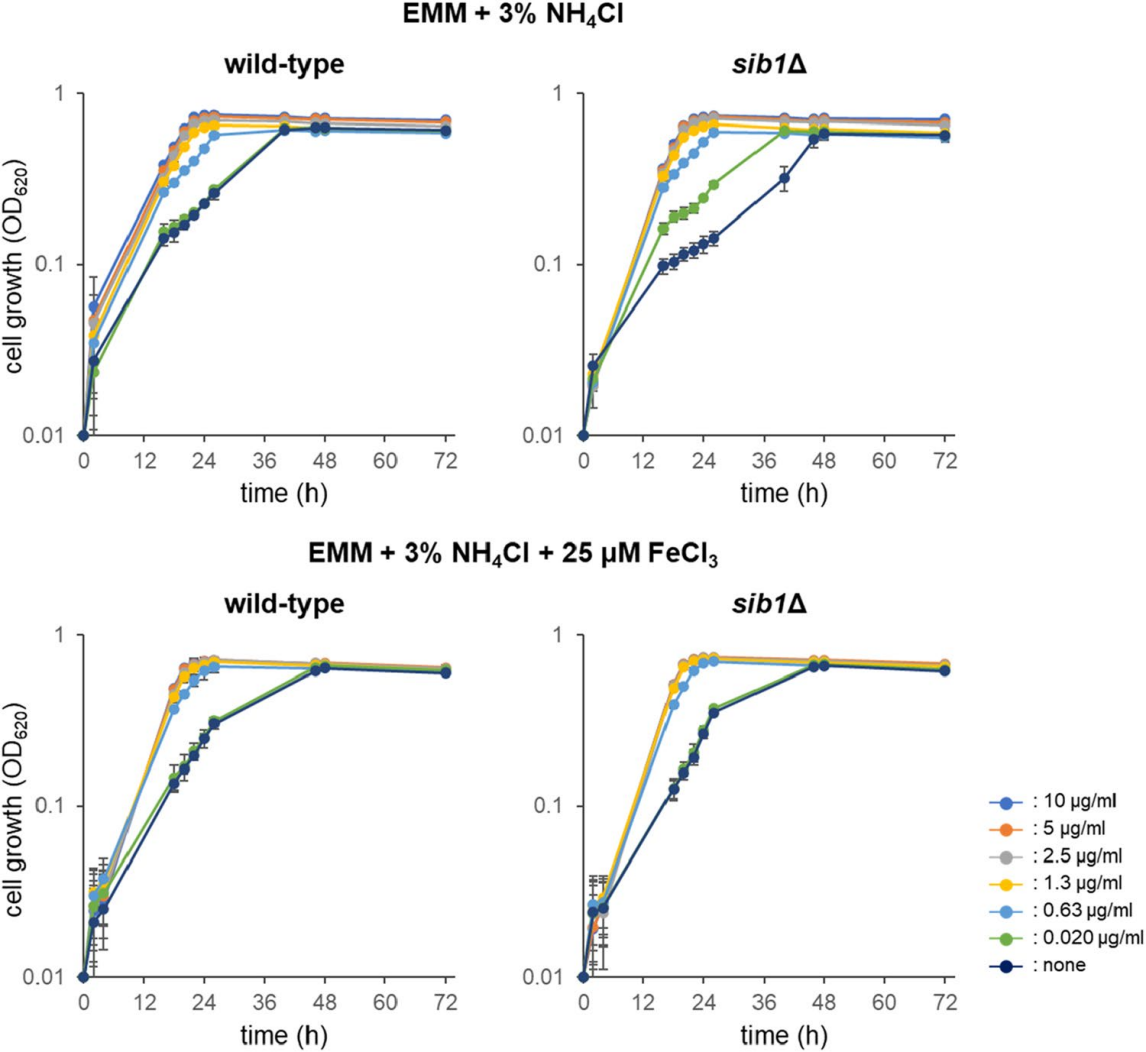
Effect of ferrichrome and inorganic iron on the cell growth in high ammonium media. Wild-type and *sib1*Δ cells were cultivated in EMM containing 3.0% NH_4_Cl supplemented with various concentrations of ferrichrome. EMM contained 0.74 µM of FeCl_3_ and 2.0% glucose. Data represent the mean ± SD (*n* = 3).

Since ferrichrome is an iron chelator, we expected that ferrichrome may function to store iron within cells. To investigate this possibility, we examined the effect of inorganic iron on the growth of the wild-type and *sib1*Δ cells (Fig. 5). Fission yeast cells have a reductive iron assimilation (RIA) system by which inorganic iron can be taken up into cells^30,31^. As expected, the difference between wild-type and *sib1*Δ cells disappeared under the iron-replete condition (supplementation with 25 μM FeCl_3_). This suggested that the mutant cells were starved for iron. However, the effect of ferrichrome (≥ 0.83 μg/ml (1.2 μM)) on the growth of the cells was still observed in the presence of FeCl_3_ (25 μM), suggesting the RIA system is not very efficient compared with the siderophore system. Alternatively, different function(s) of ferrichrome other than iron uptake may exist, such as iron transport between organelles.

Lastly, the effect of varying the glucose concentration on ferrichrome was investigated (Fig. S4). The glucose concentration affected the cell density in the stationary phase; the cell density was low when the glucose concentration was low. Ferrichrome did not affect the final cell density at 2.0% glucose, but did increase it at 1.0% or 0.5% glucose. Glucose was effectively used by cells in the presence of ferrichrome (Fig. S4). It is likely that ferrichrome increased mitochondrial activity, which enabled efficient utilization of glucose downstream of the glycolytic pathway. This phenomenon was also observed under the iron-replete condition, implying that iron uptake by siderophores is highly efficient.

In this study, we identified ferrichrome as a metabolite involved in the adaptive growth of fission yeast under high ammonium conditions. High concentrations of ammonium suppressed the growth of leucine-auxotrophic cells, which might be attributed to the suppression of leucine uptake^23^. Ferrichrome seemed to support leucine uptake since *pub1*Δ cells did not respond to ferrichrome (Fig. 2A). Additionally, *cat1*Δ cells showed higher sensitivity to high ammonium and weaker adaptive growth by ferrichrome (Fig. 2C). These results are supported by the fact that Pub1 stabilizes Cat1^25^. Ferrichrome seems to regulate the stability and/or the cellular localization of amino acid transporters including Cat1. Alternatively, *cat1*Δ cells might produce a lower amount of ferrichrome. Various possibilities for the Cat1 function should be considered. It is noted that it is unlikely that ferrichrome unlocks the NCR in general since growth suppression by glutamate, another preferred nitrogen source, was not recovered by ferrichrome (Fig. S2). Instead, ferrichrome specifically antagonized the action of ammonium.

High ammonium suppressed the growth of not only leucine-auxotrophic cells but also wild-type cells although a higher ammonium concentration was required for inhibiting the growth of wild-type cells. Time course analyses revealed that the growth suppression was observed after 14 h of exposure to high ammonium, which was more severely observed in the ferrichrome-deficient *sib1*Δ cells. Ferrichrome suppressed the effect of high ammonium in a concentration-dependent manner. Ammonium was reported to increase the pH of lysosomes in mammalian cells^32^. Recently, iron was shown to be necessary and sufficient for mammalian cell proliferation under lysosomal dysfunction that may impair iron recovery from depository protein ferritin in the lysosomes^33^. Iron did not restore lysosomal function, but instead, it reversed other cellular processes related to iron depletion. If a similar scenario is also true in fission yeast, ferrichrome may restore the iron-dependent pathways to compensate for the ammonium-induced dysfunction of vacuoles, the yeast acidic organelles. Alternatively, ferrichrome may suppress the dysfunction of vacuole induced by high ammonium by unknown mechanisms. Currently, it is unclear whether vacuolar dysfunction affects NCR in *leu1-32* cells.

Ferrichrome is mainly found in cells. The amount of ferrichrome in the culture supernatant was not changed when the ferrichrome transporter Str1 was knocked out, indicating that ferrichrome is not actively excreted out of cells. On the agar plates, ferrichrome can leak out from dead cells, which may induce adaptive growth of leucine-auxotrophic cells (Fig. 1B). From the viewpoint of the survival of species, it is reasonable to share the metabolites between dead cells and living cells to enable the survival of progenies in the population. In the natural environment, fission yeast can uptake ferrichrome that other fungal species produce and excrete.

In general, NRPS products are thought to be secondary metabolites that are synthesized using primary metabolites and are not essential for cell growth. In fission yeast, ferrichrome is an intracellular metabolite that supports the robust growth of leucine-auxotrophic and prototrophic cells under high ammonium conditions. This metabolite also contributes to efficient glucose utilization. On the basis of this work, we propose that ferrichrome is a sub-primary metabolite that reinforces primary metabolism, at least in fission yeast. Elucidation of the function of this sub-primary metabolite will not only lead to a detailed understanding of primary metabolism, but will also provide insight into the evolution of secondary metabolites.

## Materials and methods

### Yeast strains and growth media

Yeast strains and oligo DNAs used in this study are listed in Tables S1 and S2. Gene deletion mutants with leucine-auxotrophy were generated using the auxotrophic Bioneer library v5.0 by random sporulation using wild-type cells^34^. Correct gene deletion was confirmed by colony PCR. Gene deletion mutants were prepared by a PCR-based strategy^35^, replacing the entire coding region with *ura4* gene in pURA4 (see below), or deleting the ORF by CRISPR-Cas9 strategy. CRISPR-Cas9 strategy was conducted as reported previously using plasmids with some modifications^36^. First, the *Sma* I-*Sma* I region in pMZ379^36^ was cloned into the Gateway vector pDONR221, and the resultant plasmid was named pEDIT379N. This region was firstly amplified as two fragments using primer pairs of SmaI-pMZ379-F1 and pMZ379N-U1, and pMZ379N-D1 and SmaI-pMZ379-R1, and then the resultant fragments were fused using their overlapping sequences in the second PCR. Thus, pEDIT379N has the *Not* I site in place of the *Csp* CI site of pMZ379. Instead of constructing sgRNA-containing plasmids by inverse PCR as described^36^, we prepared sgRNA-containing fragments as a fusion of two PCR fragments amplified using an upstream primer SphI-F and a downstream primer SphI-R, each of which was combined with an oppositely oriented sgRNA primer. pEDIT379N linearized by restriction enzymes was used as template. The resultant PCR fragment containing the sgRNA sequence was cloned into pMZ379dS constructed by deleting the *Sph* I-*Sph* I region of pMZ379. For deletion of a gene of interest, its upstream and downstream franking regions were prepared as described^37^. mCherry was introduced into the *leu1-32* locus in some strains, which was done by using plasmid pBiD3-R25-mCherry (see below). Yeast cells were cultivated in rich yeast extract (YE) medium, consisting of 0.5% yeast extract and 2% glucose, or defined Edinburgh minimal medium 2 (EMM)^38^. Concentration of NH_4_Cl in EMM is 93.5 mM (0.5%, w/v) unless indicated. EMM-N does not contain any nitrogen source. Concentrations of supplements (adenine, uracil, isoleucine, leucine, and valine) were 0.2 mM. Ferrichome purified from the fission yeast cells (only Fig. 1) or purchased from Sigma was used. NSF was synthesized as described previously^27^.

### Plasmid construction

pURA4 was constructed by replacing the *kanMX* marker of pFA6a-kanMX6^35^ by the *ura4* gene. The *ura4* gene was amplified by PCR using the *Sac*I or *Bgl*II recognition site-containing primers ura4-BglII-F and ura4-SacI-R, digested with *Bgl*II and *Sac*I, and cloned into the pFA6a-kanMX6 digested with *Bgl*II and *Sac*I. pBiD3-R25-mCherry used for mCherry expression was constructed based on the *leu1*-tageting vector pDUAL^39^. The intergenic region between the ORFs of *leu3* and *rpl2502* was PCR-amplified using primes B2_SmaI-Prpl25-leu3 and B2_EcoRV-Pleu3-rpl25, and inserted upstream of the *ADH1* terminator in pDUAL. The intergenic region between *leu3* and *rpl2502* could function as bidirectional promoter inferred from the genome structure. We therefore inserted the terminator of *leu3* amplified by PCR using primers B1_SphI-rpl25term and B1_SmaI-rpl25term upstream of the putative *leu3*/*rpl2502* bidirectional promoter. The resultant vector pBiD3-R25 had the *leu3*/*rpl2502* promoter sandwiched between the oppositely oriented *leu3* and *ADH1* terminators. The ORF encoding mCherry was amplified by PCR using primers New_Prpl25-SmaI-mCherry-F and C2-mCherry-Rv from the mCherry expression plasmid pmCherry-N1 (TaKaRa Bio), and then inserted between the *leu3*/*rpl2502* promoter and the *ADH1* terminator by employing the gap-repair cloning technique. Thus, mCherry was expected to show constitutive expression under the regulation of the *rpl2502* promoter and the *ADH1* terminator.

### Growth assay

Adaptive growth was observed on EMM containing leucine, with different concentrations of NH_4_Cl. Cells that were freshly inoculated on agar media were suspended in sterile water at 0.2 OD_595_, spotted on the media (five spots; 3 µl/spot), and incubated at 30 °C. Wild-type cells, 50% MeOH extracts of cells (see below), NSF or ferrichrome were spotted next to the test strains. When adaptive growth of *eca39*Δ cells were tested, a ten-fold dilution series of cell suspensions was prepared with a starting concentration of 1.0 OD_595_. 3 μl of cell suspensions were spotted on solid media and incubated at 30 °C. Growth in liquid media was examined in 96 well plates. Cell suspension with 0.01 OD_595_ (50 μl) were mixed with 100 μl of media containing a variety of concentrations of ferrichrome and incubated at 30 °C. Cell growth was assessed by turbidity at 620 nm measured by Multiskan FC (Thermo scientific).

### Isolation and analyses of ferrichrome

An overnight culture of wild-type cells in EMM was inoculated on EMM plates (0.1 ml per plate) at 30 °C for 48 h. Cells were suspended in 50% MeOH (10 ml per *φ*9 cm petri dish), which was centrifuged to obtain an active extract. 50% was the optimal concentration of MeOH for efficient extraction of the active substance. In a pilot study, the extract was filtered (0.2 µm), 8 ml of which was diluted with water three times and loaded onto an SPE cartridge (C18, 500 mg, Sigma). The column was eluted by 20, 40, 60, and 100% MeOH (1 ml each). 20% and 40% MeOH fractions were found to be active. To isolate the active substance, 40 ml of the 50% MeOH extract collected from five plates was subjected to an SPE cartridge (5 g). The column was eluted by 25% MeOH. Seven fractions with each of 3 ml were collected, which were subjected to adaptive growth assay and HPLC analyses. Potent activities were detected for the 2nd to 4th fractions. In the HPLC analyses employing a reversed-phase column, a peak with maximum absorption at 425 nm was detected in the active fractions. The active fractions were subjected to HPLC to collect the characteristic peak with a maximum absorption at 425 nm. This substance was revealed to be ferrichrome by co-injection analyses (Fig. S1).

The HPLC conditions for analyses were as follows: column, 5C8-MS, *φ*4.6 × 250 mm, (Nacalai tesque); solvent system, 15% MeOH for 12 min, 15–90% MeOH in 3 min, 90% MeOH for 2 min, 90 to 15% in 3 min, followed by 15% for 3 min, with a flow rate of 1 ml/min; detection, PDA. Conditions for preparative HLPC were as follows: column, 5C8-MS (*φ*10 × 250 mm, Nacalai tesque); solvent system, 15–40% MeOH in 15 min, 40–90% in 3 min, 90–90% in 2 min, 90–15% in 2 min,15% for 3 min, with a flow rate of 3 ml/min.

To quantify the amount of ferrichrome, cells in 10 ml culture were collected by centrifugation (2500 rpm, 3 min) and suspended in water (50 μl) and MeOH (50 μl). Cell number was counted and caffeine was added to the cell suspension as an internal control. The amount of caffeine added was dependent on the cell number. Same amount of caffeine was added to the culture supernatant. Cell suspensions was mixed with CHCl_3_/MeOH (1:1, 600 μl), vortexed for 10 s three times. RO water (600 μl) was added to the mixture, which was vortexed for 10 s three times. After centrifugation (15,000 rpm, 2 min), supernatant (1 ml) was moved to a new tube. The extract was dried *in vacuo*. Culture supernatant was subjected to an ODS open column. The column was washed three volumes of RO water, then eluted with 80% MeCN. The eluate was dried *in vacuo*. The dried specimens were dissolved in 2 mM FeCl_3_ (200 μl), which was centrifuged at 13,000 for 1 min. A part of the supernatant was analyzed by ODS-HPLC: column, PEGASIL ODS SP100 (*φ*3 × 150 mm, Senshu Scientific), solvent system, 5% MeCN for 2.5 min, 5–60% in 9.5 min, and 100% for 4.5 min, with a flow rate of 1 ml/min.

## Supplementary Information

Supplementary Information.

## Data Availability

Sequences of plasmids pEDIT379N, pBiD3-R25-mCherry, and pURA4 are available at DDBJ under the accession numbers LC727554, LC727555, and LC727556, respectively. The datasets used and/or analysed during the current study available from the corresponding author on reasonable request.
