# Supplementary material for: Ferrichrome, a fungal-type siderophore, confers high ammonium tolerance to fission yeast
Source: Sci Rep. 2022 Oct 27;12:17411. doi: 10.1038/s41598-022-22108-0 (PMC9613971; doi:10.1038/s41598-022-22108-0)
Supplement: Supplementary file 1 — Supplementary Information. [file 41598_2022_22108_MOESM1_ESM.pdf]

Supplementary Information

Ferrichrome, a fungal-type siderophore, confers high ammonium tolerance to fission yeast

Authors

Po-Chang Chiu[1], Yuri Nakamura[1], Shinichi Nishimura[1,2,*], Toshitsugu Tabuchi[1], Yoko Yashiroda[3], Go Hirai[3,4], Akihisa Matsuyama[1,3], and Minoru Yoshida[1,2,3*]

1. Department of Biotechnology, Graduate School of Agricultural and Life Sciences, The University of Tokyo, Tokyo 113-8657, Japan
2. Collaborative Research Institute for Innovative Microbiology, The University of Tokyo, Tokyo 113-8657, Japan
3. RIKEN Center for Sustainable Resource Science, Saitama 351-0198, Japan
4. Graduate School of Pharmaceutical Sciences, Kyushu University, 3-1-1 Maidashi, Higashi-ku, Fukuoka 812-8582, Japan

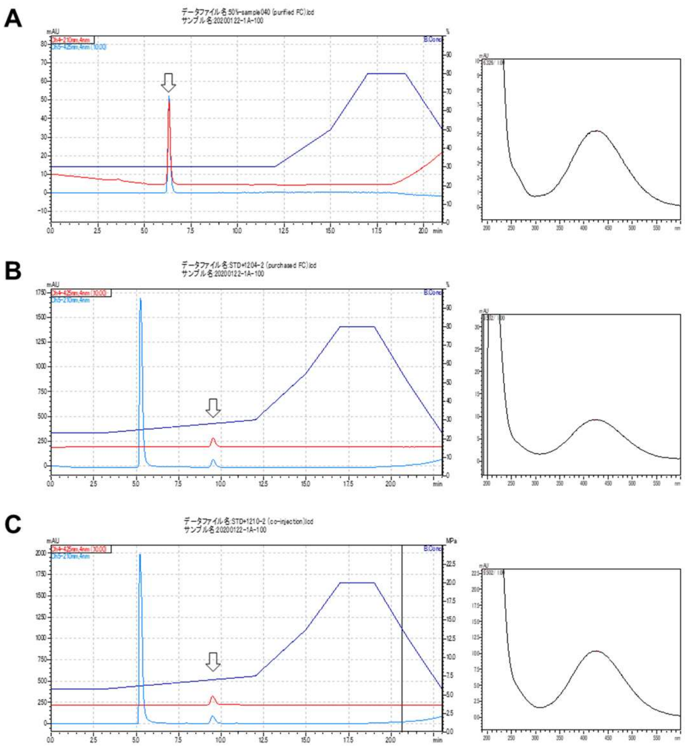

Figure S1. Co-injection analysis of purified and authentic ferrichrome.

Purified and authentic ferrichrome were analyzed by HPLC. A. Purified ferrichrome. B. Purchased ferrichrome. C. Co-injection analyses of purified and purchased ferrichomre. Chromatogram at 210 nm (light blue) and 425 nm (red) and percentage of MeOH (blue) are shown. UV/Vis spectra of the peaks with an arrow are shown in the right panels.

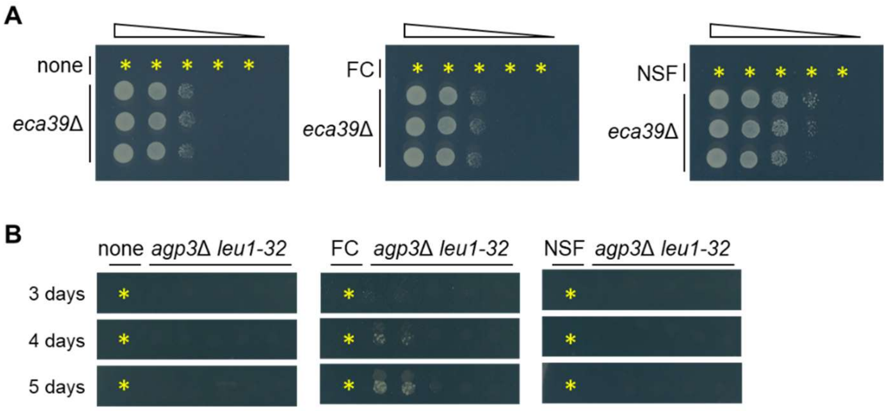

Figure S2. Different effects of ferrichrome and NSF on adaptive growth.

A: *eac39Δ ade6-M210 ura4-D18 leu1-32* cells were inoculated on media containing a high concentration of glutamate. Cell suspensions with serial dilution were spotted on EMM-N supplemented with 2.0% glutamate and 0.2 mM each of Ade, Ura, Ile, Leu, and Val, which were cultivated at 30 ^{o}C for 5 days. 50% methanol (none; 3 μl), ferrichrome (FC; 150 ng/3 μl in 50% methanol) or NSF (150 ng/3 μl in 50% methanol) was spotted at the sites with asterisk. One of representative images from three independent experiments are shown.

B. *agp3Δ leu1-32* cells were inoculated on EMM containing 2.0% NH_4Cl supplemented with 0.2 mM of leucine. Cells were cultivated at 30°C for 3, 4, and 5 days. The amounts of inducers were the same as A. One of representative images from three independent experiments are shown.

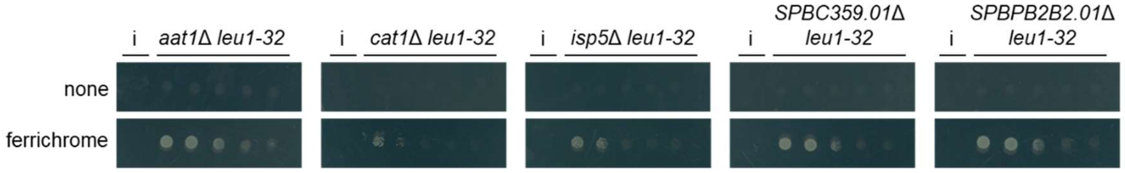

Figure S3. Growth of leucine-auxotrophic cells lacking amino acid transporters.

Cells were cultivated on EMM containing 2.0% NH_4Cl supplemented with leucine. A sample of 50% MeOH (3 µl) or ferrichrome (150 ng in 3 µl 50% MeOH) was spotted next to the yeast cell suspensions (denoted as ‘i’). Plates were incubated for three days.

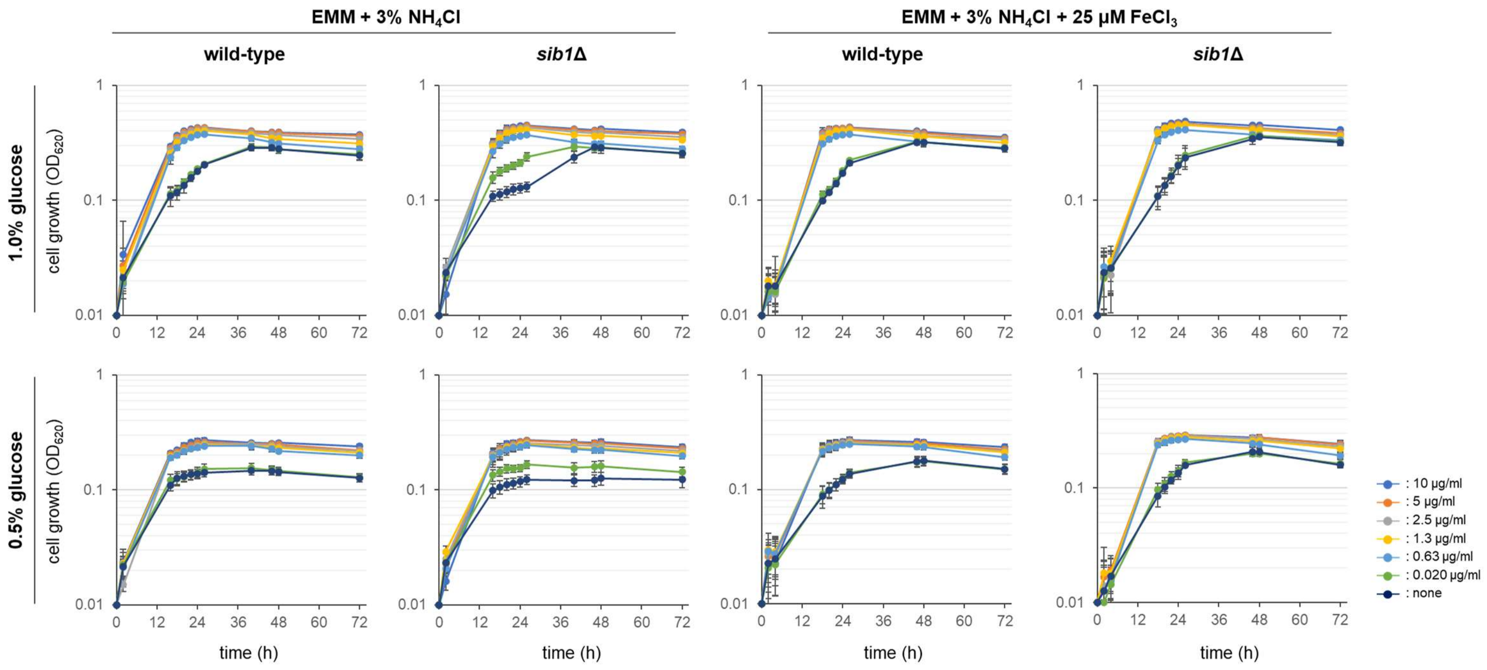

Figure S4. Effect of glucose concentrations on the cell growth of wild-type and *sib1*Δ cells. Wild-type and *sib1*Δ cells were cultivated in EMM containing 3.0% NH_4Cl and 1.0 or 0.5% glucose, supplemented with various concentrations of ferrichrome. EMM contains 0.74 μM of $FeCl_3$. Data represent the mean ± SD (n = 3).

Table S1. Fission yeast strains used in this study.

Strain	Genotype	Notes
JY1	h^-	Lab stock
TN4	h^- *leu1-32*	Lab stock
TB010	h^+ *agp3::kanMX6 leu1-32*	This study
TB035	h^+ *pub1::kanMX6 leu1-32*	This study
PC014	*str1* Δ *leu1-32*	This study
PC018	*sib1::kanMX6 leu1-32*	This study
PC036	h^- *any1:: kanMX6 leu1-32*	This study
TB169	h^- *aat1:: kanMX6 leu1-32*	This study
TB139	h^+ *SPBPB2B2.01::kanMX6 leu1-32*	This study
TB134	h^+ *SPBC359.01:: kanMX6 leu1-32*	This study
TB154	h^- *isp5::kanMX6 leu1-32*	This study
TB174	*cat1:: kanMX6 leu1-32*	This study
PC060	h^- *cat1::ura4*$^+$ *ura4-D18 leu1-32*	This study
V5-P36-66	h^+ *eca39::kanMX6 ade6-M216 ura4-D18 leu1-32*	Bioneer ver 5.0
YN6	h^- *leu1*$^+$ << *pBID3-R25-mCherry*	This study
YN23	h^- *sib1* Δ (CRISPR) *leu1*$^+$ << *pBID3-R25mCherry*	This study
YN31	h^- *str1* Δ (CRISPR) *leu1*$^+$ << *pBID3-R25-mCherry*	This study

Table S2. Oligo DNAs used in this study.

Name	Sequence	Purpose
ura4-BglII-F	GGGGTCTAGATCTACAAATCCCACTGGCTATATG	construction of pURA4
ura4-SacI-R	GGGGAATTCGAGCTCGTTTAAACGTGATTTTTATCTTG	construction of pURA4
B2_SmaI-Prpl25-leu3	TGGCCACTGCAGCCCGGGCTTGACTCTATAGTGTAAAAG	construction of pBiD3-R25-mCherry
B2_EcoRV-Pleu3-rpl25	AGTGGCGCGCCGGATCCGATATCGGAGACTGTAGCGATTTT	construction of pBiD3-R25-mCherry
B1_SphI-rpl25term	GATTACGCCAAGCTTGCATGCATTACTTGCTATTATCTTAAC	construction of pBiD3-R25-mCherry
B1_SmaI-rpl25term	CGGGCTGCAGTGGCCACTTAAAAACTAGTTTAATAA	construction of pBiD3-R25-mCherry
New_Prpl25-SmaI-mCherry-F	CTATAGAGTCAAGCCCGGGATGGTGAGCAAGGGCGAGGAG	construction of pBiD3-R25-mCherry
C2-mCherry-Rv	CGCGCCGGATCCGATCTACTTGTACAGCTCGTCCATG	construction of pBiD3-R25-mCherry
SmaI-pMZ379-F1	GGGGACAAGTTTGTACAAAAAAGCAGGCTACCGGTACCGCACCAGTGTC	construction of pEDIT379N
SmaI-pMZ379-R1	GGGGACCACTTTGTACAAGAAAGCTGGGATTACGAATTCGAGCTCGGTAC	construction of pEDIT379N
pMZ379N-U1	CCGAAGAAGCGGCCGCGTTTTAGAGCTAGAAATAGCA	construction of pEDIT379N
pMZ379N-D1	TCTAAAACGCGGCCGCTTCTTCGGTACAGGTTATGTT	construction of pEDIT379N
SphI-F	AGCAGGCGCTCTACATGAGCATG	gene deletion by CRISPR Cas9
SphI-R	TTGCTTATGTTGGTGGTAGTTGGC	gene deletion by CRISPR Cas9
str1-sgFw1	AGTACCGTTACACCCATCGG-GTTTTAGAGCTAGAAATAGCAAG	gene deletion by CRISPR Cas9
str1-sgRv1	CCGATGGGTGTAACGGTACT-TTCTTCGGTACAGGTTATGTTTTTTG	gene deletion by CRISPR Cas9
str1-HRFw	TTTAATTTAGCTATTTTAAATTTCTGCATTTCTATATTGTTTTCCCAAGAAAATCATATG	gene deletion by CRISPR Cas9
str1-HRRv	AATATCTGCCGAAATGCGAATTACGATTTTTATTATCTCCCATATGATTTTCTTGGGAAA	gene deletion by CRISPR Cas9
str1-CkFw	TTTCCATTGTAACATCCCTGGC	gene deletion by CRISPR Cas9
str1-CkRv	GAACTTCGTGAAAGGCGGTG	gene deletion by CRISPR Cas9
sib1-sgFw1	TTGGAATAACGGTCTGACGT-GTTTTAGAGCTAGAAATAGCAAG	gene deletion by CRISPR Cas9
sib1-sgRv1	ACGTCAGACCGTTATTCCAA-TTCTTCGGTACAGGTTATGTTTTTTG	gene deletion by CRISPR Cas9
sib1'-HRRv	TTTATCATATAATTGTTTATAGGTCCTTCATAGCAATAAAGTCGTTGGAT-AAGTCTCAAT	gene deletion by CRISPR Cas9
sib1'-HRFw	ACCTTCGTTATAAAATATTTCCGTGTCACTAAATTTTTTGATTGAGACTTAT-CCAACGAC	gene deletion by CRISPR Cas9
sib1-CkRv	AACGAACTTTAAGGCTGCGC	gene deletion by CRISPR Cas9
sib1'-CkRv	TCACCACTTGAGCCAATGGG	gene deletion by CRISPR Cas9
cat1-F1	ATACATACTAAATTGCTTTTAGCGC	gene deletion
cat1-F2	TTAATTAACCCGGGGATCCG-TTTGCAGATACCACCAGTACAA	gene deletion
cat1-R2	GTTTAAACGAGCTCGAATTCATC-AATCGATGAAATTTCCTTGAGT	gene deletion
cat1-R1	TACACGACATATTAACTGTCCTA	gene deletion
cat1-S2	TCTCACCAGCAAGATATACTTT	confirmation of gene deletion
cat1-S3	ATTAGGGGAAATGCTAATGCAGACC	confirmation of gene deletion
